# Supplementary material for: Oxidized glutathione reverts carbapenem resistance in blaNDM-1-carrying Escherichia coli
Source: EMBO Mol Med. 2024 Apr 2;16(5):2. doi: 10.1038/s44321-024-00061-x (PMC11099006; doi:10.1038/s44321-024-00061-x)
Supplement: Supplementary file 1 — Appendix [file 44321_2024_61_MOESM1_ESM.pdf]

Appendix

Oxidized glutathione reverts carbapenem resistance in *bla*$_{NDM-1}$-carrying *Escherichia coli*

Dongyang Ye[1,2], Xiaowei Li[1], Liang Zhao[1], Saiwa Liu[1], Xixi Jia[1], Zhinan Wang[1], Jingjing Du[1], Lirui Ge[1], Jianzhong Shen[1,*], Xi Xia[1,*]

[1]National Key Laboratory of Veterinary Public Health and Safety, College of Veterinary Medicine, China Agricultural University, Beijing, China

[2]College of Veterinary Medicine, Northwest A&F University, Yangling, Shaanxi, China

*Correspondence author: Tel.: +86-10-62732802; Fax: +86-10-62731201.

Email: sjz@cau.edu.cn (J. Shen); xxia@cau.edu.cn (X. Xia)

Table of Contents

Appendix Figure S1..3

Appendix Figure S2..5

Appendix Table S1...7

Appendix Table S2...9

Appendix Table S3...11

Appendix Table S4...13

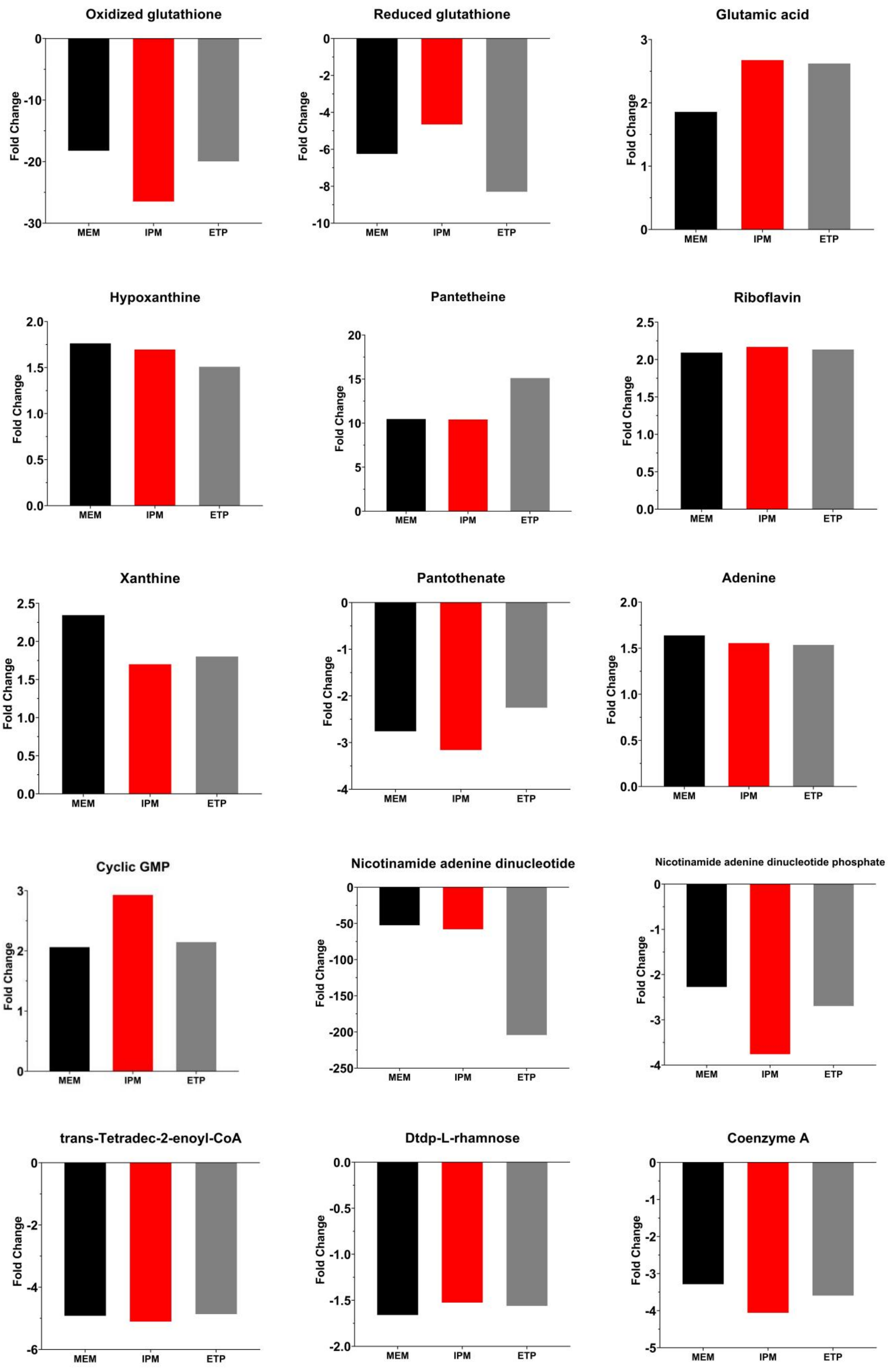

Oxidized glutathione
Fold Change
0
-10
-20
-30
MEM
IPM
ETP
Reduced glutathione
Fold Change
0
-2
-4
-6
-8
-10
MEM
IPM
ETP
Glutamic acid
Fold Change
3
2
1
0
MEM
IPM
ETP
Hypoxanthine
Fold Change
2.0
1.5
1.0
0.5
0.0
MEM
IPM
ETP
Pantetheine
Fold Change
20
15
10
5
0
MEM
IPM
ETP
Riboflavin
Fold Change
2.5
2.0
1.5
1.0
0.5
0.0
MEM
IPM
ETP
Xanthine
Fold Change
2.5
2.0
1.5
1.0
0.5
0.0
MEM
IPM
ETP
Pantothenate
Fold Change
0
-1
-2
-3
-4
MEM
IPM
ETP
Adenine
Fold Change
2.0
1.5
1.0
0.5
0.0
MEM
IPM
ETP
Cyclic GMP
Fold Change
3
2
1
0
MEM
IPM
ETP
Nicotinamide adenine dinucleotide
Fold Change
0
-50
-100
-150
-200
-250
MEM
IPM
ETP
Nicotinamide adenine dinucleotide phosphate
Fold Change
0
-1
-2
-3
-4
MEM
IPM
ETP
trans-Tetradec-2-enoyl-CoA
Fold Change
0
-2
-4
-6
MEM
IPM
ETP
Dtdp-L-rhamnose
Fold Change
0.0
-0.5
-1.0
-1.5
-2.0
MEM
IPM
ETP
Coenzyme A
Fold Change
0
-1
-2
-3
-4
-5
MEM
IPM
ETP

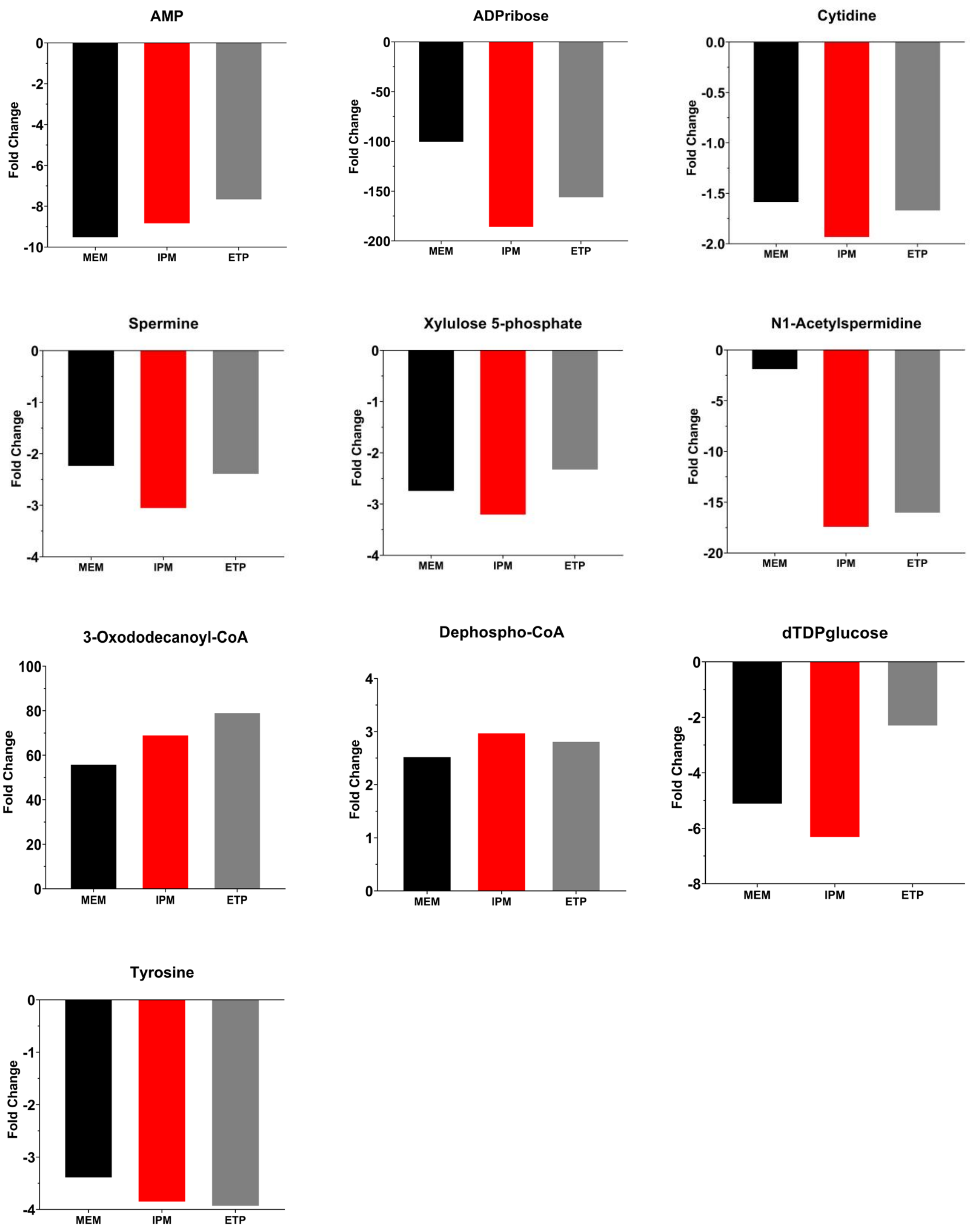

Appendix Figure S1. Common differential metabolites induced by three carbapenems.

Metabolites with a positive fold change are upregulated, while those with a negative fold change are downregulated.

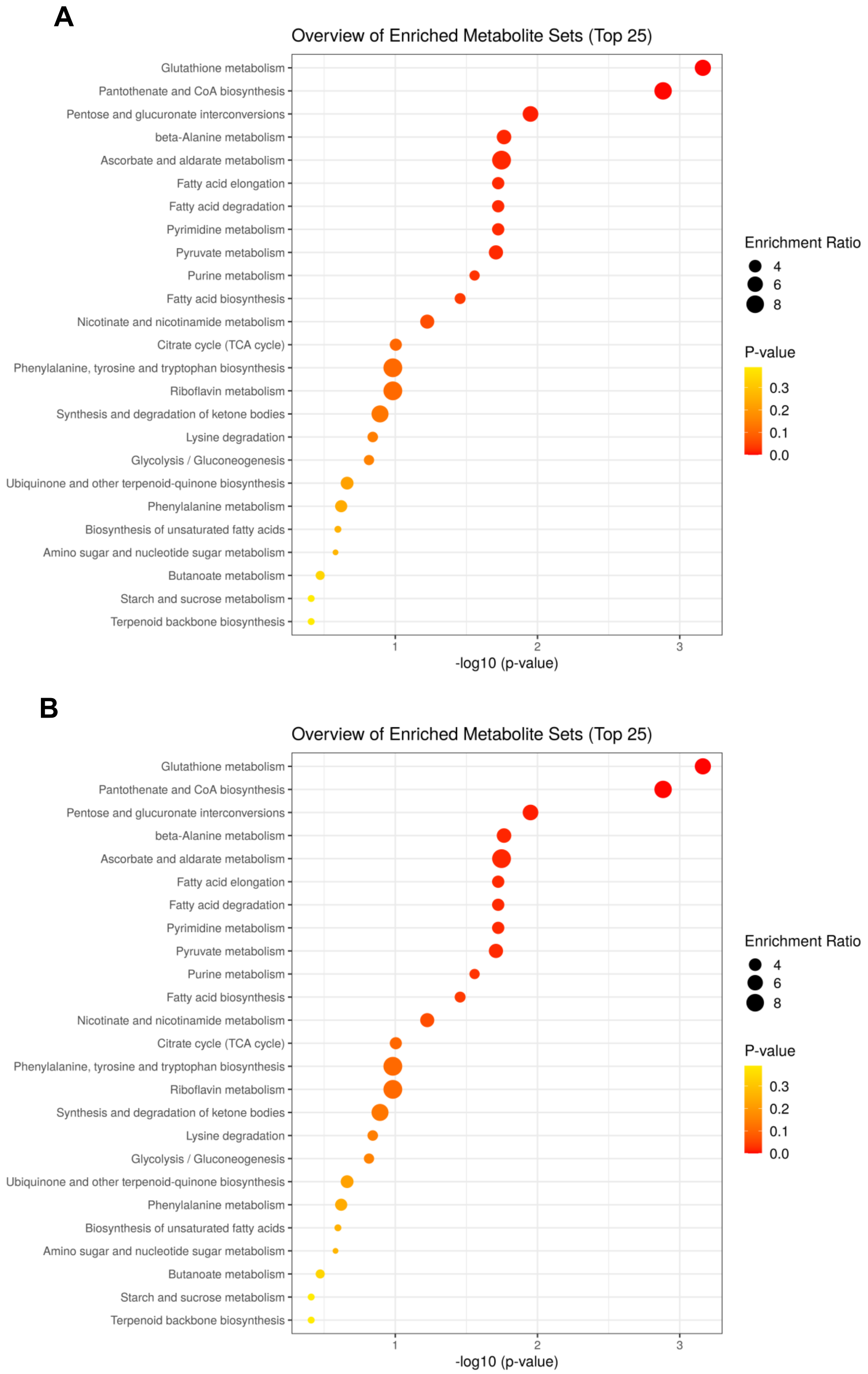

A
Overview of Enriched Metabolite Sets (Top 25)
Glutathione metabolism
Pantothenate and CoA biosynthesis
Pentose and glucuronate interconversions
beta-Alanine metabolism
Ascorbate and aldarate metabolism
Fatty acid elongation
Fatty acid degradation
Pyrimidine metabolism
Pyruvate metabolism
Purine metabolism
Fatty acid biosynthesis
Nicotinate and nicotinamide metabolism
Citrate cycle (TCA cycle)
Phenylalanine, tyrosine and tryptophan biosynthesis
Riboflavin metabolism
Synthesis and degradation of ketone bodies
Lysine degradation
Glycolysis / Gluconeogenesis
Ubiquinone and other terpenoid-quinone biosynthesis
Phenylalanine metabolism
Biosynthesis of unsaturated fatty acids
Amino sugar and nucleotide sugar metabolism
Butanoate metabolism
Starch and sucrose metabolism
Terpenoid backbone biosynthesis
1
2
3
-log10 (p-value)
Enrichment Ratio
4
6
8
P-value
0.3
0.2
0.1
0.0
B
Overview of Enriched Metabolite Sets (Top 25)
Glutathione metabolism
Pantothenate and CoA biosynthesis
Pentose and glucuronate interconversions
beta-Alanine metabolism
Ascorbate and aldarate metabolism
Fatty acid elongation
Fatty acid degradation
Pyrimidine metabolism
Pyruvate metabolism
Purine metabolism
Fatty acid biosynthesis
Nicotinate and nicotinamide metabolism
Citrate cycle (TCA cycle)
Phenylalanine, tyrosine and tryptophan biosynthesis
Riboflavin metabolism
Synthesis and degradation of ketone bodies
Lysine degradation
Glycolysis / Gluconeogenesis
Ubiquinone and other terpenoid-quinone biosynthesis
Phenylalanine metabolism
Biosynthesis of unsaturated fatty acids
Amino sugar and nucleotide sugar metabolism
Butanoate metabolism
Starch and sucrose metabolism
Terpenoid backbone biosynthesis
1
2
3
-log10 (p-value)
Enrichment Ratio
4
6
8
P-value
0.3
0.2
0.1
0.0

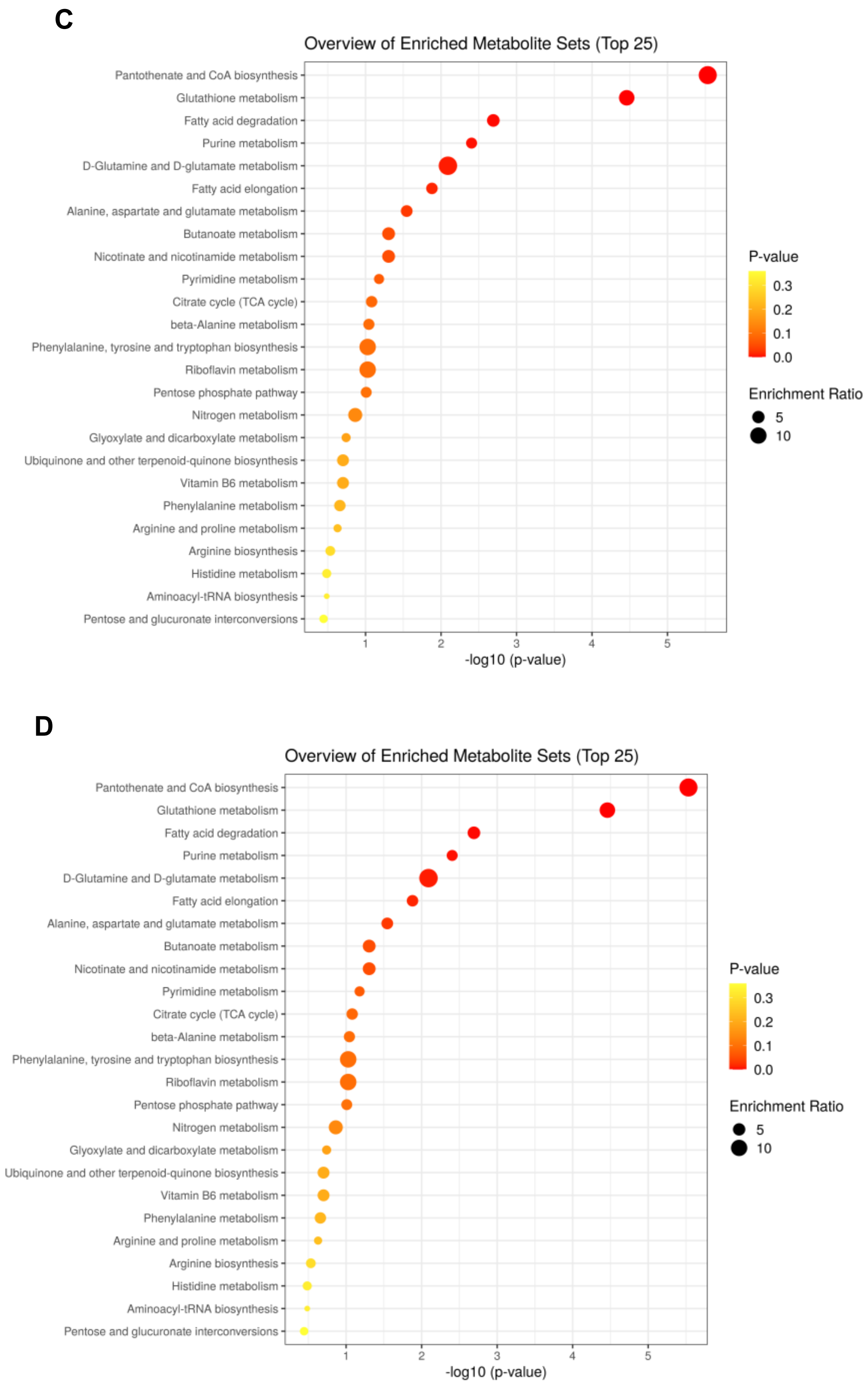

Appendix Figure S2. Metabolic pathway analysis of *bla*$_{\text{NDM-1}}$-carrying *E.coli*.
(A) Metabolic perturbations induced by *bla*$_{\text{NDM-1}}$. (B) Metabolic perturbations of *bla*$_{\text{NDM-1}}$-carrying *E.coli* induced by meropenem. (C) Metabolic perturbations of *bla*$_{\text{NDM-1}}$-carrying *E.coli* induced by imipenem. (D) Metabolic perturbations of *bla*$_{\text{NDM-1}}$-carrying *E.coli* induced by ertapenem.

Appendix Table S1. Differential metabolites of *bla*NDM-1-carrying *E.coli* under meropenem stimulation

Compound	Formula	Compound ID
**Upregulated**		
Cyclic GMP	$C_{10}H_{12}N_5O_7P$	C06194
3-Oxododecanoyl-CoA	$C_{33}H_{56}N_7O_{18}P_3S$	C05263
5-Methylthioadenosine	$C_{11}H_{15}N_5O_3S$	C00170
Adenine	$C_5H_5N_5$	C00147
cAMP	$C_{10}H_{12}N_5O_6P$	C00575
Decanoic acid	$C_{10}H_{20}O_2$	C01571
Dephospho-CoA	$C_{21}H_{35}N_7O_{13}P_2S$	C00882
Dihydrouracil	$C_4H_6N_2O_2$	C00429
Glyceryl phosphate	$C_3H_9O_6P$	C03189
Hypoxanthine	$C_5H_4N_4O$	C00262
Glutamic acid	$C_5H_9NO_4$	C00025
Niacin	$C_6H_5NO_2$	C00253
Palmitic Acid	$C_{16}H_{32}O_2$	C00249
Pantetheine 4'-phosphate	$C_{11}H_{23}N_2O_7PS$	C01134
Riboflavin	$C_{17}H_{20}N_4O_6$	C00255
Thymidine	$C_{10}H_{14}N_2O_5$	C00214
Xanthine	$C_5H_4N_4O_2$	C00385
Xanthosine	$C_{10}H_{12}N_4O_6$	C01762
Xanthosine 5-triphosphate	$C_{10}H_{15}N_4O_{15}P_3$	HMDB0000293
**Downregulated**		
Pantothenate	$C_9H_{17}NO_5$	C00864
ADP	$C_{10}H_{15}N_5O_{10}P_2$	C00008
ADPribose	$C_{15}H_{23}N_5O_{14}P_2$	C06743
AMP	$C_{10}H_{14}N_5O_7P$	C00020
Coenzyme A	$C_{21}H_{36}N_7O_{16}P_3S$	C00010
Cytidine	$C_9H_{13}N_3O_5$	C00475
dTDP-4-acetamido-4,6-dideoxy-D-galactose	$C_{18}H_{29}N_3O_{15}P_2$	HMDB0012222
dTDP-4-dehydro-6-deoxy-D-glucose	$C_{16}H_{24}N_2O_{15}P_2$	C11907
dTDPglucose	$C_{16}H_{26}N_2O_{16}P_2$	HMDB0001328
Dtdp-L-rhamnose	$C_{16}H_{26}N_2O_{15}P_2$	C03319
Guanosine	$C_{10}H_{13}N_5O_5$	C00387
Leucine	$C_6H_{13}NO_2$	C00123
Tyrosine	$C_9H_{11}NO_3$	C00082
N1,N12-Diacetylspermine	$C_{14}H_{30}N_4O_2$	C03413
N1-Acetylspermidine	$C_9H_{21}N_3O$	C00612

NAD	$C_{21}H_{27}N_7O_{14}P_2$	C00003
NADP	$C_{21}H_{28}N_7O_{17}P_3$	C00006
Oxidized glutathione	$C_{20}H_{32}N_6O_{12}S_2$	C00127
Phosphoenolpyruvic acid	$C_3H_5O_6P$	C00074
Reduced glutathione	$C_{10}H_{17}N_3O_6S$	C00051
Ribose 1-phosphate	$C_5H_{11}O_8P$	C00620
Spermine	$C_{10}H_{26}N_4$	C00750
trans-Dodec-2-enoyl-CoA	$C_{33}H_{56}N_7O_{17}P_3S$	C03221
trans-Tetradec-2-enoyl-CoA	$C_{35}H_{60}N_7O_{17}P_3S$	C05273
UDPglucose	$C_{15}H_{24}N_2O_{17}P_2$	C00029
UDP-N-acetylmuramate	$C_{20}H_{31}N_3O_{19}P_2$	C01050
Xylulose 5-phosphate	$C_5H_{11}O_8P$	C00231

Appendix Table S2. Differential metabolites of *bla*NDM-1-carrying *E.coli* under imipenem stimulation

Compound	Formula	Compound ID
**Upregulated**		
Pantetheine	$C_{11}H_{22}N_2O_4S$	C00831
Cyclic GMP	$C_{10}H_{12}N_5O_7P$	C06194
2-Oxo-3-hydroxy-4-phosphobutanoate	$C_4H_7O_8P$	C06054
3-Oxododecanoyl-CoA	$C_{33}H_{56}N_7O_{18}P_3S$	C05263
Adenine	$C_5H_5N_5$	C00147
Deoxyribose	$C_5H_{10}O_4$	C01801
Dephospho-CoA	$C_{21}H_{35}N_7O_{13}P_2S$	C00882
Dihydrouracil	$C_4H_6N_2O_2$	C00429
Elaidic Acid	$C_{18}H_{34}O_2$	C01712
Hypoxanthine	$C_5H_4N_4O$	C00262
Glutamic acid	$C_5H_9NO_4$	C00025
Palmitic Acid	$C_{16}H_{32}O_2$	C00249
Pantetheine 4'-phosphate	$C_{11}H_{23}N_2O_7PS$	C01134
Riboflavin	$C_{17}H_{20}N_4O_6$	C00255
Xanthine	$C_5H_4N_4O_2$	C00385
Xanthosine	$C_{10}H_{12}N_4O_6$	C01762
**Downregulated**		
Pantothenate	$C_9H_{17}NO_5$	C00864
ADP	$C_{10}H_{15}N_5O_{10}P_2$	C00008
ADPribose	$C_{15}H_{23}N_5O_{14}P_2$	C06743
AMP	$C_{10}H_{14}N_5O_7P$	C00020
Citric acid	$C_6H_8O_7$	C00158
Coenzyme A	$C_{21}H_{36}N_7O_{16}P_3S$	C00010
Cytidine	$C_9H_{13}N_3O_5$	C00475
D-Alanyl-D-alanine	$C_6H_{12}N_2O_3$	C00993
dTDP-4-acetamido-4,6-dideoxy-D-galactose	$C_{18}H_{29}N_3O_{15}P_2$	HMDB0012222
dTDPglucose	$C_{16}H_{26}N_2O_{16}P_2$	C00842
Dtdp-L-rhamnose	$C_{16}H_{26}N_2O_{15}P_2$	C03319
Tyrosine	$C_9H_{11}NO_3$	C00082
N1-Acetylspermidine	$C_9H_{21}N_3O$	C00612
NAD	$C_{21}H_{27}N_7O_{14}P_2$	C00003
NADP	$C_{21}H_{28}N_7O_{17}P_3$	C00006
Oxidized glutathione	$C_{20}H_{32}N_6O_{12}S_2$	C00127
Reduced glutathione	$C_{10}H_{17}N_3O_6S$	C00051
Spermine	$C_{10}H_{26}N_4$	C00750
Succinic acid	$C_4H_6O_4$	C00042

trans-Dodec-2-enoyl-CoA	$C_{33}H_{56}N_7O_{17}P_3S$	C03221
trans-Tetradec-2-enoyl-CoA	$C_{35}H_{60}N_7O_{17}P_3S$	C05273
UMP	$C_9H_{13}N_2O_9P$	C00105
Xylulose 5-phosphate	$C_5H_{11}O_8P$	C00231

Appendix Table S3. Differential metabolites of *bla*NDM-1-carrying *E.coli* under ertapenem stimulation

Compound	Formula	Compound ID
**Upregulated**		
Pantetheine	$C_{11}H_{22}N_2O_4S$	C00831
Cyclic GMP	$C_{10}H_{12}N_5O_7P$	C06194
3-Oxododecanoyl-CoA	$C_{33}H_{56}N_7O_{18}P_3S$	C05263
Adenine	$C_5H_5N_5$	C00147
Dephospho-CoA	$C_{21}H_{35}N_7O_{13}P_2S$	C00882
GDP	$C_{10}H_{15}N_5O_{11}P_2$	C00035
Glyceryl phosphate	$C_3H_9O_6P$	C03189
Hypoxanthine	$C_5H_4N_4O$	C00262
Inosine	$C_{10}H_{12}N_4O_5$	C00294
Glutamic acid	$C_5H_9NO_4$	C00025
N-Acetylserine	$C_5H_9NO_4$	HMDB0002931
Niacin	$C_6H_5NO_2$	C00253
Pantetheine 4'-phosphate	$C_{11}H_{23}N_2O_7PS$	C01134
Riboflavin	$C_{17}H_{20}N_4O_6$	C00255
Xanthine	$C_5H_4N_4O_2$	C00385
**Downregulated**		
Pantothenate	$C_9H_{17}NO_5$	C00864
ADPribose	$C_{15}H_{23}N_5O_{14}P_2$	C06743
AMP	$C_{10}H_{14}N_5O_7P$	C00020
Coenzyme A	$C_{21}H_{36}N_7O_{16}P_3S$	C00010
Cytidine	$C_9H_{13}N_3O_5$	C00475
Cytosine	$C_4H_5N_3O$	C00380
D-Alanyl-D-alanine	$C_6H_{12}N_2O_3$	C00993
`dTDP-4-dehydro-6-deoxy-D-glucose	$C_{16}H_{24}N_2O_{15}P_2$	C11907
dTDPglucose	$C_{16}H_{26}N_2O_{16}P_2$	HMDB0001328
Dtdp-L-rhamnose	$C_{16}H_{26}N_2O_{15}P_2$	C03319
FAD	$C_{27}H_{33}N_9O_{15}P_2$	C00016
Glycine betaine	$C_5H_{11}NO_2$	C00719
Guanosine	$C_{10}H_{13}N_5O_5$	C00387
Carnitine	$C_7H_{15}NO_3$	C00318
Leucine	$C_6H_{13}NO_2$	C00123
Tyrosine	$C_9H_{11}NO_3$	C00082
N1,N12-Diacetylspermine	$C_{14}H_{30}N_4O_2$	C03413
N1-Acetylspermidine	$C_9H_{21}N_3O$	C00612
NAD	$C_{21}H_{27}N_7O_{14}P_2$	C00003
NADP	$C_{21}H_{28}N_7O_{17}P_3$	C00006
Oxidized glutathione	$C_{20}H_{32}N_6O_{12}S_2$	C00127

Reduced glutathione	$C_{10}H_{17}N_3O_6S$	C00051
Spermine	$C_{10}H_{26}N_4$	C00750
Succinic acid	$C_4H_6O_4$	C00042
Thiamine diphosphate	$C_{12}H_{18}N_4O_7P_2S$	C00068
trans-Tetradec-2-enoyl-CoA	$C_{35}H_{60}N_7O_{17}P_3S$	C05273
UDPglucose	$C_{15}H_{24}N_2O_{17}P_2$	C00029
UDP-N-acetylmuramate	$C_{20}H_{31}N_3O_{19}P_2$	C01050
Xylulose 5-phosphate	$C_5H_{11}O_8P$	C00231

Appendix Table S4. The primer sequences used in the qRT-PCR analysis

Primer	Primer sequence (5'-3')	Annealing temperature (°C)	Sequence length (bp)
bla$_{NDM}$-qRT-F	TGGCAGCACACTTCCTATCTC	60	212
bla$_{NDM}$-qRT-R	ATACCGCCCATCTTGTCCTG		
16S rRNA-qRT-F	CGGTGAATACGTTCYCGG	60	143
16S rRNA-qRT-R	GGWTACCTTGTTACGACTT		
