# Supplementary figures and images for: Oxidized glutathione reverts carbapenem resistance in blaNDM-1-carrying Escherichia coli
Source: EMBO Mol Med. 2024 Apr 2;16(5):2. doi: 10.1038/s44321-024-00061-x (PMC11099006; doi:10.1038/s44321-024-00061-x)

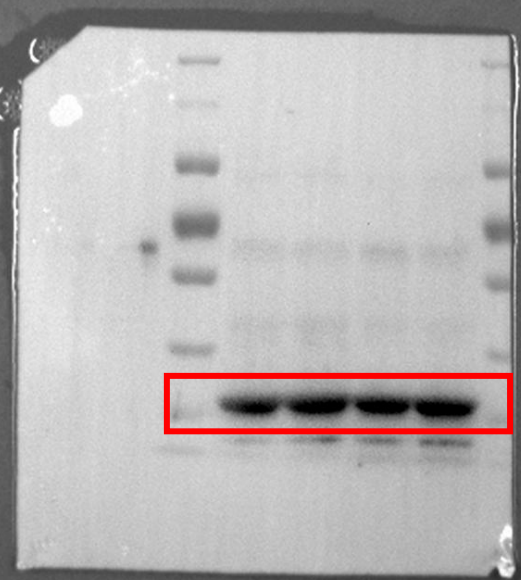

← GAPDH

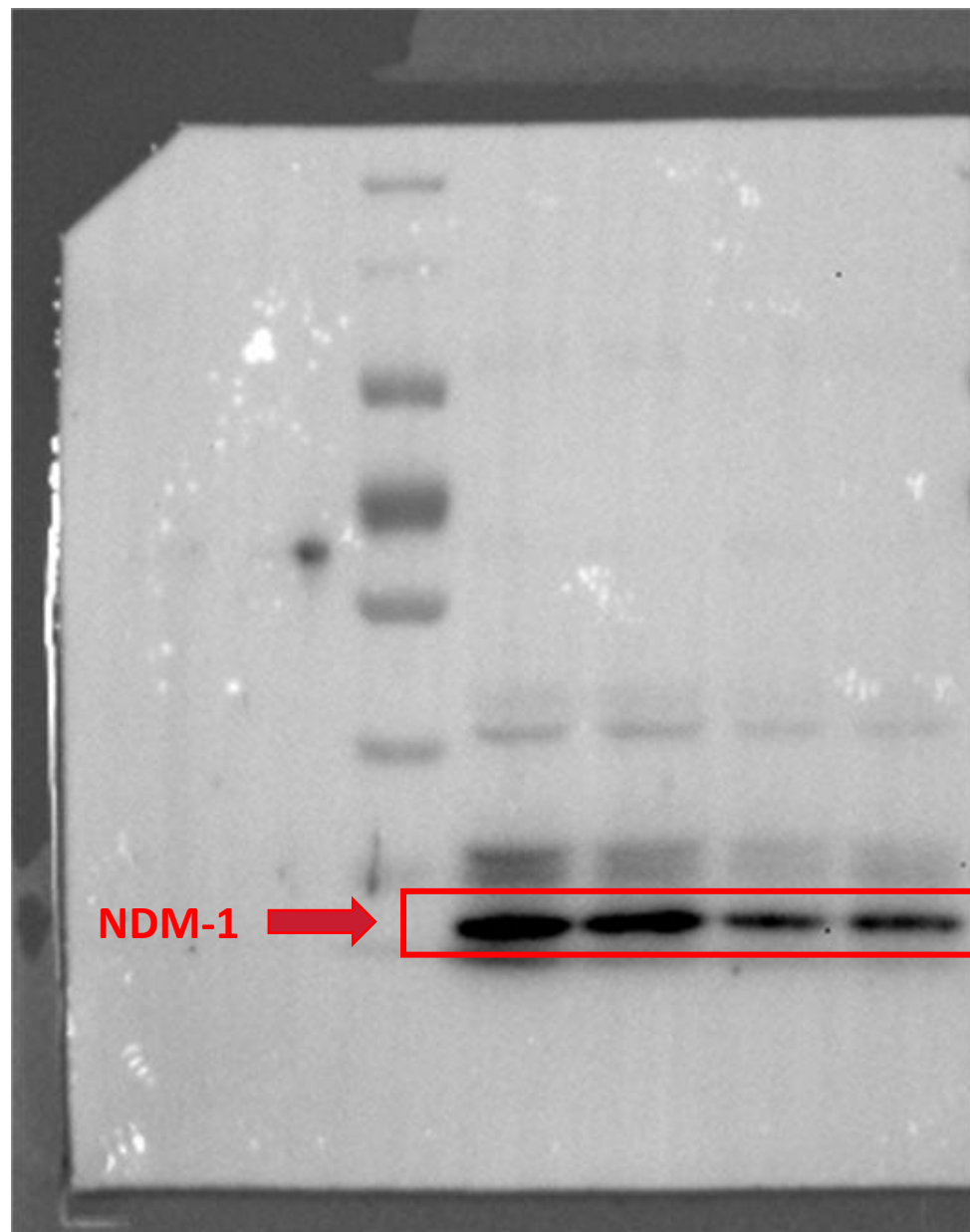

NDM-1 →

Supplement: Supplementary file 3 — Source data Fig. 2 [file 44321_2024_61_MOESM3_ESM.zip › Figure 2-1/Figure 2G/Figure 2G.pdf]

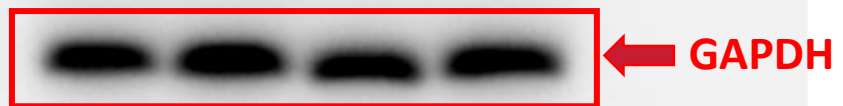

NDM-1

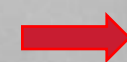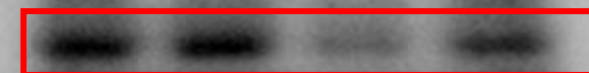

Supplement: Supplementary file 3 — Source data Fig. 2 [file 44321_2024_61_MOESM3_ESM.zip › Figure 2-1/Figure 2H/Figure 2H.pdf]
