# Supplementary material for: Oxidized glutathione reverts carbapenem resistance in blaNDM-1-carrying Escherichia coli
Source: EMBO Mol Med. 2024 Apr 2;16(5):2. doi: 10.1038/s44321-024-00061-x (PMC11099006; doi:10.1038/s44321-024-00061-x)
Supplement: Supplementary file 5 — Expanded View Figures [file 44321_2024_61_MOESM5_ESM.pdf]

Expanded View Figures

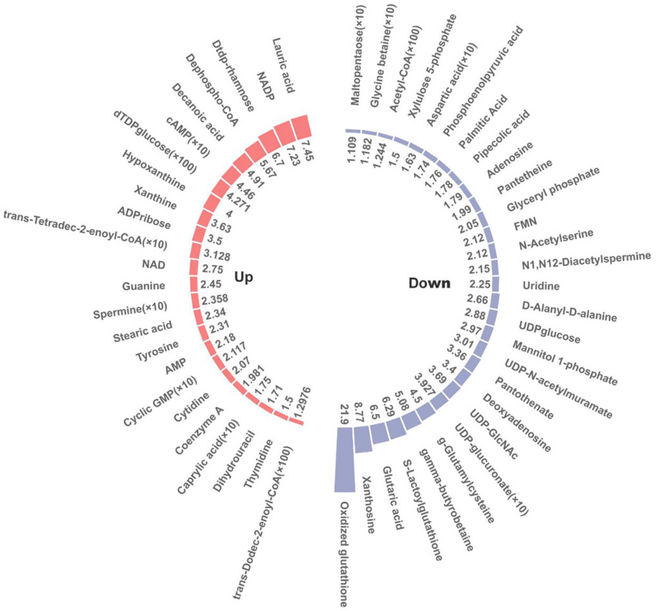

Figure EV1. ***bla*$_{\text{NDM-1}}$ induces broad metabolic alterations in *E. coli.***

Metabolomics analysis of *E. coli* carrying pHSG398 and pHSG398/*bla*$_{\text{NDM-1}}$ was performed using liquid chromatography-high resolution mass spectrometry. Upregulated and downregulated metabolites in drug-resistant strain are indicated with red and blue, respectively.

 © The Author(s)

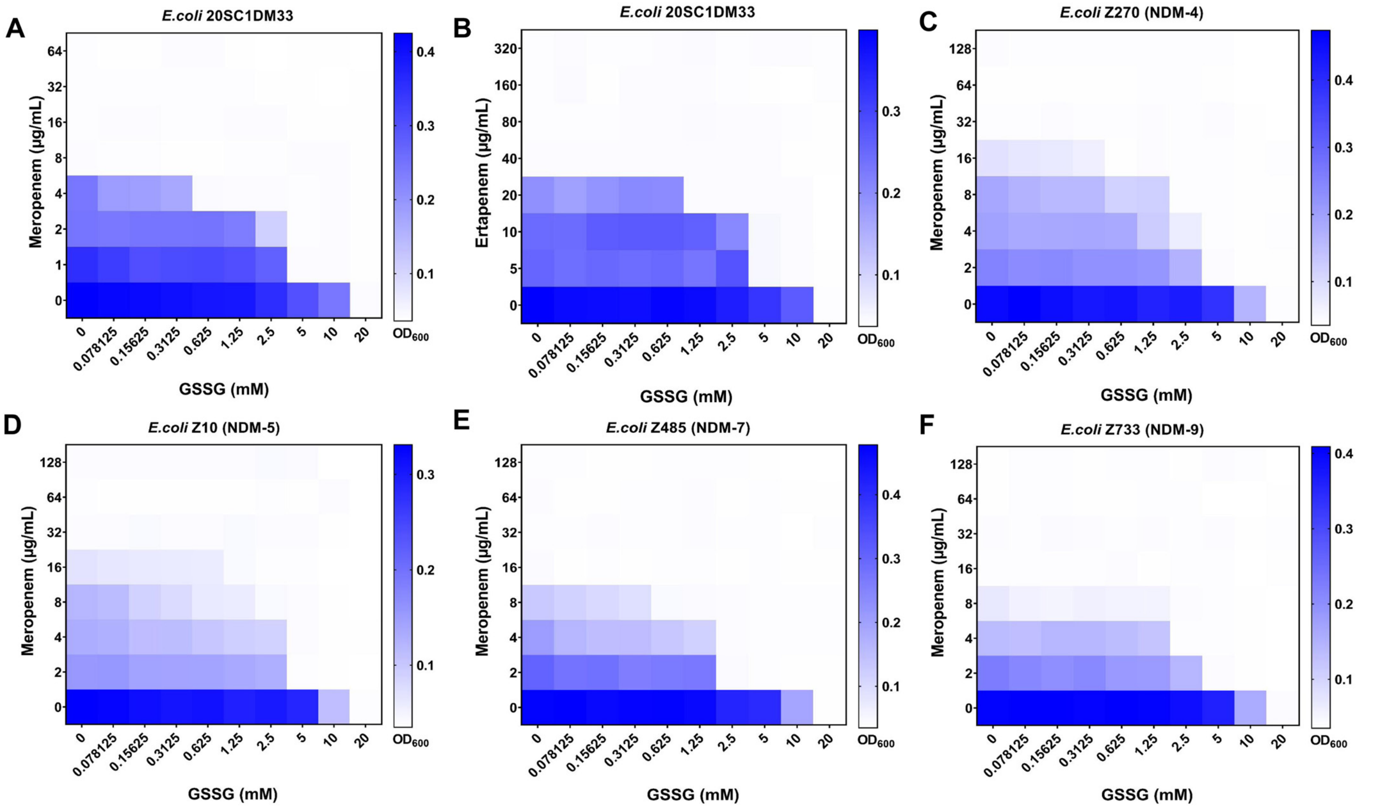

Figure EV2. Synergistic effect of GSSG combined with carbapenems on wild-type resistant strains *E. coli* carrying *bla*$_{\mathrm{NDM-1}}$ and variants.

(**A**) *E. coli* 20SC1DM33 treated with meropenem and GSSG. (**B**) *E. coli* 20SC1DM33 treated with ertapenem and GSSG. (**C**) *E. coli* Z270 carrying *bla*$_{\mathrm{NDM-4}}$ treated with meropenem and GSSG. (**D**) *E. coli* Z10 carrying *bla*$_{\mathrm{NDM-5}}$ treated with meropenem and GSSG. (**E**) *E. coli* Z485 carrying *bla*$_{\mathrm{NDM-7}}$ treated with meropenem and GSSG. (**F**) *E. coli* Z733 carrying *bla*$_{\mathrm{NDM-9}}$ treated with meropenem and GSSG.

© The Author(s)

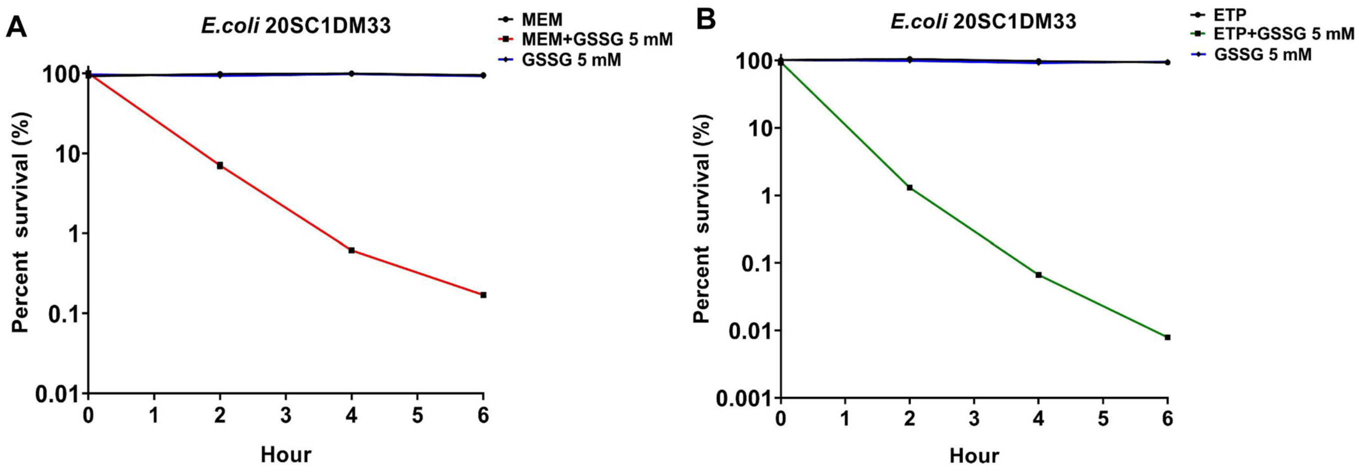

Figure EV3. In vitro synergistic bactericidal effect of oxidized glutathione (GSSG) in combination with carbapenems on a wild-type strain *E. coli*.

(**A**) Survival of *E. coli* 20SC1DM33 after treatment of GSSG, meropenem (MEM), and combination therapy ($n = 3$ biological replicates). (**B**) Survival of *E. coli* 20SC1DM33 after treatment of GSSG, ertapenem (ETP), and combination therapy ($n = 3$ biological replicates).

 © The Author(s)

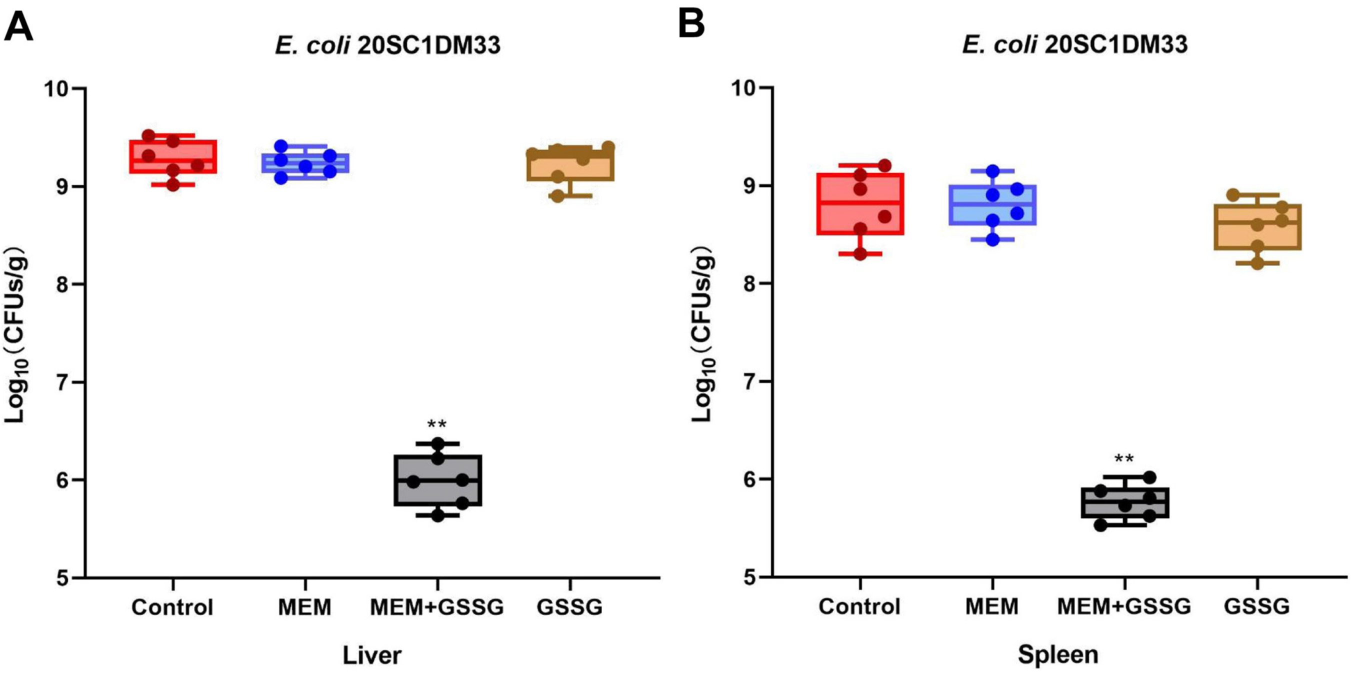

Figure EV4. Bacterial loads in the tissues of mice infected with a wild-type resistant strain *E. coli.*

(**A**) Bacterial loads in the liver of infected mice ($n = 6$ per group) after treatment of oxidized glutathione (GSSG), meropenem (MEM), and combination therapy. (**B**) Bacterial loads in the spleen of infected mice ($n = 6$ per group) after treatment of GSSG, MEM, and combination therapy. Data Information: Box plots represent the median with interquartile range, and the whiskers indicate the minimum and maximum values. One-way ANOVA analysis, $^{**}p < 0.01$.

© The Author(s)

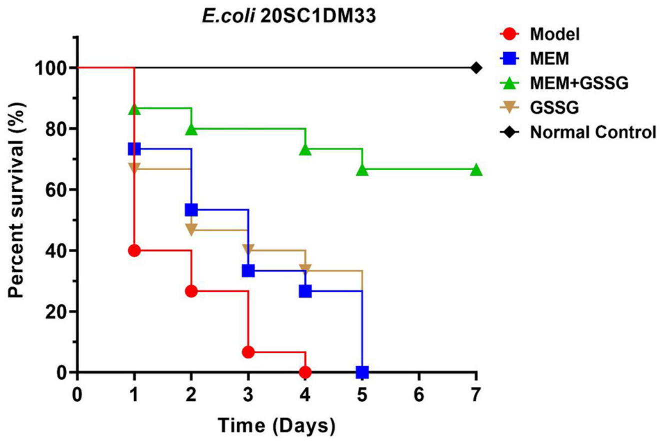

Figure EV5. In vivo synergistic bactericidal effect of oxidized glutathione (GSSG) in combination with meropenem (MEM) on a wild-type strain *E. coli*.

Survival plot of infected mice ($n = 15$ per group) after treatment of GSSG, MEM, and combination therapy.

 © The Author(s)
